# Paper-Based Magneto-Resistive Sensor: Modeling, Fabrication, Characterization, and Application

**DOI:** 10.3390/s18124392

**Published:** 2018-12-11

**Authors:** Meriem Akin, Autumn Pratt, Jennifer Blackburn, Andreas Dietzel

**Affiliations:** 1 Institute of Microtechnology, Department of Mechanical Engineering, Braunschweig University of Technology, 38124 Braunschweig, Germany; a.dietzel@tu-braunschweig.de; 2 Sibley School of Mechanical and Aerospace Engineering, Cornell University, Ithaca, NY 14853, USA; agp76@cornell.edu; 3 School of Mechanical Engineering, Purdue University, West Lafayette, IN 47907, USA; jenniferbrooke93@gmail.com

**Keywords:** paper-based, magneto-resistance, sensor

## Abstract

In this work, we developed and fabricated a paper-based anisotropic magneto-resistive sensor using a sputtered permalloy (Ni${}_{81}$Fe${}_{19}$) thin film. To interpret the characteristics of the sensor, we proposed a computational model to capture the influence of the stochastic fiber network of the paper surface and to explain the physics behind the empirically observed difference in paper-based anisotropic magneto-resistance (AMR). Using the model, we verified two main empirical observations: (1) The stochastic fiber network of the paper substrate induces a shift of $45^{\circ }$ in the AMR response of the paper-based Ni${}_{81}$Fe${}_{19}$ thin film compared to a Ni${}_{81}$Fe${}_{19}$ film on a smooth surface as long as the fibrous topography has not become buried. (2) The ratio of magnitudes of AMR peaks at different anisotropy angles and the inverted AMR peak at the $90^{\circ }$-anisotropy angle are explained through the superposition of the responses of Ni${}_{81}$Fe${}_{19}$ inheriting the fibrous topography and smoother Ni${}_{81}$Fe${}_{19}$ on buried fibrous topographies. As for the sensitivity and reproducibility of the sensor signal, we obtained a maximum AMR peak of 0.4%, min-max sensitivity range of [0.17,0.26]%, average asymmetry of peak location of 2.7 $\frac{\mathrm{kA}}{m}$ within two consecutive magnetic loading cycles, and a deviation of 250–850 $\frac{A}{m}$ of peak location across several anisotropy angles at a base resistance of ∼100 Ω. Last, we demonstrated the usability of the sensor in two educational application examples: a textbook clicker and interactive braille flashcards.

## 1. Introduction

### 1.1. State of the Art

Due to its low cost, biodegradability, and renewability, among other properties, paper is being investigated as a functional material for disruptive applications such as interactive surfaces [1], skin-like sensing [2], rapid prototyping of electronics [3], computing [4] and many others. The mathematics of origami and kirigami, contemporized with laser cutting ([5,6]), enable non-planar paper products such as pop-ables [7], foldable circuit boards [8] and three-dimensional actuators ([9,10]). Here, actuation of three-dimensional paper structures was enabled by employing magnetism as an added value to the light weight and mechanical bendability of paper, e.g., by embedding magnetic ferrites during or after papermaking ([11,12]) or by depositing thick layers of organic resins filled with magnetic particles ([13,14]).

In our previous work ([15,16]), we considered the magnetic properties of non-patterned, thin films of permalloy (a ferromagnetic alloy made of 81% of nickel and 19% of iron; termed Ni${}_{81}$Fe${}_{19}$ in the remaining of this paper) sputtered on paper. In particular, we found that the magnetic coercivity of thin films of Ni${}_{81}$Fe${}_{19}$ on paper substrates, i.e., the sensor resistance to an external magnetic field decreases with an increase in film thickness. In this paper, motivated by the aforementioned observation, we address the magneto-resistive properties of paper-based thin films of Ni${}_{81}$Fe${}_{19}$ and the feasibility of a paper-based sensor using the principle of anisotropic magneto-resistance (AMR).

Conventionally, AMR sensors and Ni${}_{81}$Fe${}_{19}$ thin films are applied on smooth rigid substrates in planar patterns. As an attempt to understand paper-based AMR, we consider similar artificially engineered geometries. First, in the era of analog recording, a common practice was to cover the Ni${}_{81}$Fe${}_{19}$ coating by equally spaced, oblique conductive strips, so-called barber poles [17], to direct the current flow within the Ni${}_{81}$Fe${}_{19}$ film along the anisotropy angle of $45^{\circ }$, the angle formed by the nominal direction of the electrical current and the magnetic field. Second, zigzag-shaped Ni${}_{81}$Fe${}_{19}$ grooves were used to direct, and hence, to detect the presence of magnetic particles [18] for bio-sensor applications. Last, three-dimensional ferromagnetic nano-wire networks with a nominal shape anisotropy along a $\pm 45^{\circ }$-angle were prepared by electro-chemical deposition into a polymeric mold [19]. Nevertheless, the impact of interfacial roughness on the magneto-resistance of thin films was mainly studied for surface roughnesses at the atomic scale (below several tens of $\overset{˚}{A}$) and for non-fibrous surfaces ([20,21]).

### 1.2. Anticipated Study

To develop a paper-based AMR sensor, we proceed as follows: First, we develop a computational model to understand the empirically observed AMR response of thin Ni${}_{81}$Fe${}_{19}$ films deposited on paper substrates. Based on these findings, we design and develop a paper-based magneto-resistive sensor that we fabricate and characterize. Last, we embed the resulting sensor in two illustrative application scenarios, where paper is already in use and the paper sensor can be naturally embedded.

**Figure 1:**
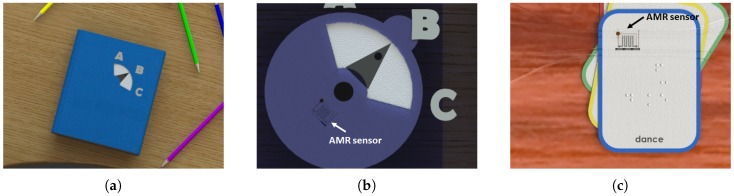
Visionary models of the use of textbook-embedded educational clickers in the classroom (**a**,**b**), and interactive braille flashcards (**c**) which could employ the paper-based AMR sensor.

As a first example of application of the sensor, we consider educational clickers that are used to engage the classroom in the teaching and learning experience. With clickers, teachers can conveniently collect answers to a question from all students in real-time and subsequently use the immediate feedback of the classroom to improve the learning experience. With the intention of adding intelligent components to paper-based educational material, for example, we propose to employ the paper-based AMR sensor as the core of an educational clicker that can be naturally embedded in a textbook. We envision a simple, tactile clicker that especially young primary school students can easily operate. When answering a question, a student can choose between three answers ‘A’, ‘B’ and ‘C’ that correspond to the distinct angular positions ‘$90^{\circ }$’, ‘$45^{\circ }$’ and ‘$0^{\circ }$’ of the AMR sensor (Figure 1a,b).

As an attempt to provide beginners to the braille system with teaching material that could help them improve their reading skills, we propose an educational game based on interactive braille flashcards as another application of the sensor (Figure 1c). A flashcard bearing a randomly oriented embossed word is given to a braille student who must properly orient the card so that the given word is in the correct reading direction. The paper-based AMR sensor embedded into the flashcard determines the orientation of the card. By means of audible or vibro-tactile indications, the sensor information is reported to the student while the flashcard is being turned. Once the reading direction corresponding to a distinct angular position of the sensor is found, the student is informed through a distinct locator tone.

While designing both applications, we consulted with education researchers who recommended the use of motor cognition such as the turning of the clicker or the flashcard. Here, the principle of AMR naturally fulfills this requirement.

## 2. Results and Discussion

### 2.1. Empirical and Computational Investigation of Paper-Based Magneto-Resistance of Ni${}_{81}$Fe${}_{19}$ Thin Film

To understand the response of the paper-based sensor, we first considered the qualitative AMR response of a 10 mm × 1 mm—strip of a Ni${}_{81}$Fe${}_{19}$ thin film sputter deposited on a paper substrate (Section 3.1). In [16], we studied the magnetic properties of paper-based thin films of Ni${}_{81}$Fe${}_{19}$ of thicknesses up to 900 nm. We found that films of 900 nm thickness exhibited the least magnetic coercivity, which we verified in this work to lead to a better AMR sensitivity. Hence, we adopt a thin film of 900 nm thickness through the entire paper. While the strip pattern enables the nominal direction of the electrical current with respect to the magnetic field, we chose the size of the strip based on two criteria: (i) feasibility of patterning during sputter deposition using a shadow mask and (ii) the ratio of the strip width to the maximum pore diameter of the paper surface. For strip widths below 1 mm, we observed the paper-based Ni${}_{81}$Fe${}_{19}$ thin films to become non-conductive.

**Figure 2:**
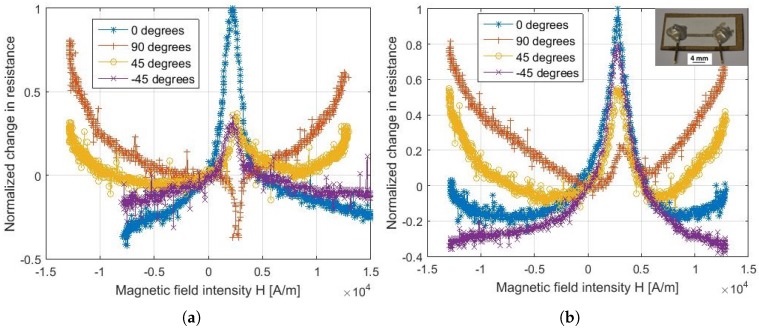
Alternating current (AC) component of magneto-resistance of 10 × 1 mm${}^{2}$-strip of Ni${}_{81}$Fe${}_{19}$ on a paper substrate (**b**) in comparison to a reference 10×1 mm${}^{2}$-strip of Ni${}_{81}$Fe${}_{19}$ on a highly smooth, fused silica substrate (**a**) for the anisotropy angles $\alpha^{AMR}\in \left\{-45^{\circ },0^{\circ },45^{\circ },90^{\circ }\right\}$. The magneto-resistance is scaled into the range [−0.5,1].

According to the characterization method presented in Section 3.3, we observed the alternating current (AC) electrical resistances of the paper-based Ni${}_{81}$Fe${}_{19}$ strip for four in-plane anisotropy angles, $\alpha^{AMR}\in \left\{-45^{\circ },0^{\circ },45^{\circ },90^{\circ }\right\}$, formed by the direction of the electrical current and the magnetic field. Figure 2a,b qualitatively contrast the AC AMR of Ni${}_{81}$Fe${}_{19}$ in the film plane deposited on a smooth fused silica substrate and a fibrous paper substrate, respectively. Both thin films exhibit a pronounced change of electrical resistance, illustrated by a peak at small intensities of the magnetic field, for the four anisotropy angles considered. The inversion of angular sensitivity that is typically known for Ni${}_{81}$Fe${}_{19}$ thin films of thicknesses above 100 nm is observed here. In particular, the highest sensitivity, usually expected at the angular configuration $\alpha^{AMR}=\frac{\pi }{2}$, is occurring at the angular configuration $\alpha^{AMR}=0$. This inversion in angular response is justified by the large coating thickness (>100 nm) and the low sputter deposition rate of Ni${}_{81}$Fe${}_{19}$ that are employed in this work, as similarly investigated in [22]. Furthermore, two major differences between the two substrates are observed: (i) the ascending order of change of magneto-resistance for the anisotropy angles considered and (ii) the opposite polarity of the peak corresponding to the anisotropy angle $90^{\circ }$. We hypothesize that the observed difference in the magneto-resistive response is biased by the stochastic fiber network of the paper surface.

**Figure 3:**
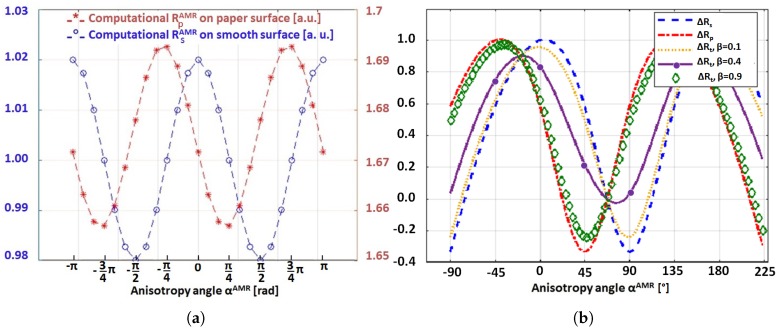
(**a**) Magneto-resistive response of a Ni${}_{81}$Fe${}_{19}$ coating on a completely fibrous paper surface, $R_{p}^{AMR}$ at β=1, compared to the response of a Ni${}_{81}$Fe${}_{19}$ coating on a smooth surface, $R_{s}^{AMR}$ at β=0. The change of magneto-resistance is expressed in arbitrary units. (**b**) Resulting %-change of AMR in dependence of the anisotropy angle for β∈{0.1,0.4,0.9}.

Employing the electro-magnetic computational model presented in Section 3.2, we investigate the impact of the interfacial fibrous nature of the paper substrate on the AMR response of a Ni${}_{81}$Fe${}_{19}$ coating. We present in Figure 3a the magneto-resistances, $R_{p}^{AMR}$ and $R_{s}^{AMR}$, obtained from the model for the simple cases of fibrous coating (β=1) and fully buried paper structure (β=0), respectively, and given in arbitrary units as a function of the anisotropy angle $\alpha^{AMR}$. We mainly observe that $R_{p}^{AMR}$ exhibits an angular shift, Γ, of $45^{\circ }$ with respect to $R_{s}^{AMR}$, similarly as if barber poles are applied on the Ni${}_{81}$Fe${}_{19}$ coating. Varying the coating geometry and the maximum stochastic fiber length, $l_{i}^{\max}$, in the model, we observe the shift of $45^{\circ }$ remaining unaltered.

Now that we know the existence of the $45^{\circ }$-shift, we explicitly invoke the $45^{\circ }$-shift in the superposition model for $R_{t}^{AMR}\left(\alpha^{AMR},\beta \right)$ proposed in Section 3.2 in order to study the AMR responses for 0<β<1. In particular, we expect that the fibrous regions of the coating to exhibit an AMR response according to:

(1) $$R_{p}^{AMR}\left(\alpha^{AMR}\right)=R_{p}^{⊥}+\left(R_{p}^{∥}-R_{p}^{⊥}\right)\cos^{2}\left(\alpha^{AMR}+\Gamma \right)=R_{p}^{⊥}+\left(R_{p}^{∥}-R_{p}^{⊥}\right)\cos^{2}\left(\alpha^{AMR}+45^{\circ }\right).$$

We also propose that the angular shift between $R_{s}$ and $R_{t}$ changes dependently on the coating thickness *t*, and hence β, due to the formation of coated regions that are independent of the fiber orientation and paper porosity. Therefore, we expect that the shift decreases with increasing *t*, and hence decreasing β, which we confirmed based on the computational results presented in Figure 3b.

In the following, we particularly consider the case of β=0.4, which we find reflects the thickness *t* of 900 nm, to interpret the measurements shown in Figure 2b. First, the observed inversion in the peak gradient for the anisotropy angle $90^{\circ }$, when compared to Figure 2a, is confirmed by the computed positive %-change of AMR. Furthermore, the ascending order of the peak magnitudes given in Figure 2b coincides with the ascending order of the computed %-changes of AMR; the peak magnitudes are marked with filled dots on the AMR curve of β=0.4 in Figure 3b. Hence, we confirmed the hypothesis that the inherited fibrous nature of the Ni${}_{81}$Fe${}_{19}$ thin film is the cause of the distinctive differences in the in-plane AMR response of paper-based Ni${}_{81}$Fe${}_{19}$. Yet, we point out that the quantitative differences cannot be captured by the current model. Due to the out-of-plane orientation of the interfacial fibers, we expect the in-plane, absolute %-change of paper-based AMR to be reduced by an out-of-plane component and, hence, to be less than the in-plane, absolute %-change of AMR on smooth substrates. Next, we empirically study the absolute %-change of paper-based AMR.

### 2.2. Sensor Characteristics

**Figure 4:**
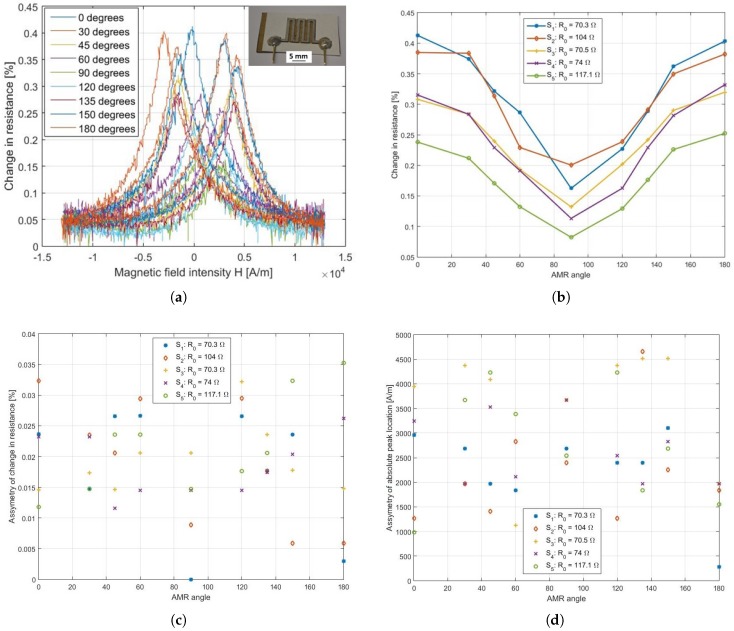
Change of resistance in dependence on (**a**) the magnetic field intensity for AMR sensor $S_{1}$, and (**b**) the angular configuration $\alpha^{AMR}$ for all five AMR sensors. Sweeping asymmetry of AMR peak magnitude (**c**) and absolute peak location (**d**) for the five AMR sensors $S_{1}$, $S_{2}$, $S_{3}$, $S_{4}$ and $S_{5}$ at representative arbitrary snapshots in time during continuous measurement.

We scrutinized the AMR performance of a set of five sensors, $P^{m}$ = {$S_{1}$, $S_{2}$, $S_{3}$, $S_{4}$ and $S_{5}$}, with close base resistances, i.e., $R_{0}$ = {70.3, 104, 70.5, 74, 117.1} Ω, for the anisotropy angles $\alpha^{AMR}=\left\{0,30,45,60,90,120,135,150,180\right\}$ degrees. While the magnitude of AMR sensitivity slightly differs from one sensor to another, which is due to the quality of the Ni${}_{81}$Fe${}_{19}$ thin film, all five AMR sensors exhibited a distinct sensitivity for each angular configuration, α + kπ, where k ∈{0,1,2,3,…}. In summary, we obtained a maximum AMR sensitivity of ≈0.4 % (Figure 4), whereas the max-to-min sensitivity range lies within [0.17,0.26]%.

We assessed the reproducibility of the AMR response for each system and for each angular configuration over consecutive magnetic loading periods. Sweeping from positive to negative magnetic fields and back, we investigate the magnitude of the AMR peak of the paper-based Ni${}_{81}$Fe${}_{19}$ meanders and the shift from the 0-magnetic field. For an anisotropy angle $\alpha^{AMR}$, we consider two distinct magnetic loading cycles, $C_{1}$ and $C_{2}$, where

(2) $${\frac{dR}{R_{0}}}_{\alpha^{AMR}}^{C_{i}}=f\left(H\right)$$

is the AMR effect in dependence on the magnetic field intensity, *H*, and for the corresponding loading cycle $C_{i}$. For the ascending loading cycle, $C_{1}$, let the magnitude and location of the AMR peak be

(3) $$\max_{H=H_{C_{1}}}{\frac{dR}{R_{0}}}_{\alpha^{AMR}}^{C_{1}}\left(H\right)$$

and

(4) $$H_{C_{1}}^{\alpha^{AMR}},$$

respectively. Similarly, for the descending loading cycle, let the magnitude and the location of the AMR peak be

(5) $$\max_{H=H_{C_{2}}}{\frac{dR}{R_{0}}}_{\alpha^{AMR}}^{C_{2}}\left(H\right)$$

and

(6) $$H_{C_{2}}^{\alpha^{AMR}},$$

respectively. Hence, we consider the asymmetry of peak magnitude such as:

(7) $$A_{m}^{\alpha^{AMR}}=\left|\max_{H=H_{C_{1}}}{\frac{dR}{R_{0}}}_{\alpha^{AMR}}^{C_{1}}\left(H\right)-\max_{H=H_{C_{2}}}{\frac{dR}{R_{0}}}_{\alpha^{AMR}}^{C_{2}}\left(H\right)\right|.$$

Additionally, we measure the asymmetry of absolute peak location as follows:

(8) $$A_{l}^{\alpha^{AMR}}=|H_{C_{1}}^{\alpha^{AMR}}\left|-\right|H_{C_{2}}^{\alpha^{AMR}}|.$$

The scatter plots given in Figure 4c,d illustrate the range of reproducibility of the AMR response of the sensor set. While the asymmetry of peak magnitude is below 10% of the maximum AMR sensitivity, the asymmetry of absolute peak location is 2.7$\frac{\mathrm{kA}}{m}$ on average for all angular configurations. For all sensors, we found that the absolute peak locations of all angular configurations deviate from each other in the range of 250–850 $\frac{A}{m}$. These measures dictate the minimum range of magnetic field intensity that is necessary to operate the sensor.

Increasing the base resistance with the sensor stack to ∼1 kΩ, as described in Section 3.1, do not change sensitivity and reproducibility.

### 2.3. Application Prototypes

**Figure 5:**
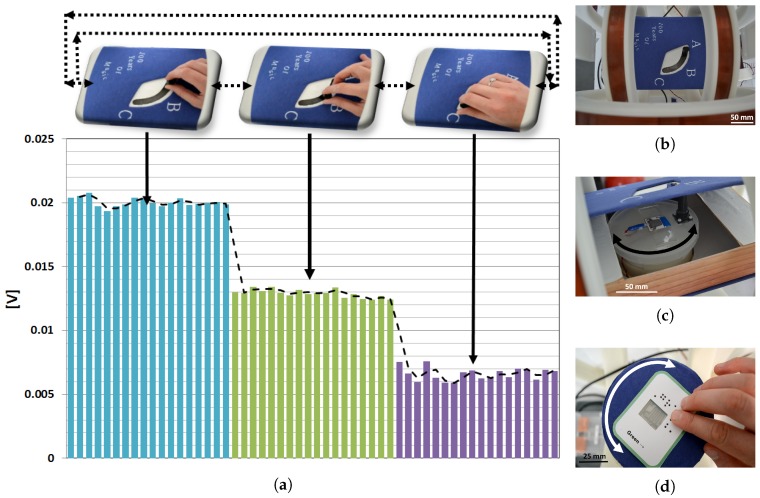
Photographs of the hybrid clicker prototype (**b**) embedded in a textbook format that houses the turnstile, the paper-based AMR sensor and a turning handle (**c**), and hybrid interactive braille flashcards prototype based on the paper-based AMR sensor and the turnstile as a work base (**d**). (**a**) shows the amplified voltage data of the AMR sensor collected during calibration. The dashed (–) line represents the rolling average over the angular responses.

Based on the set-up described in Section 3.4, and due to the lack of off-the-shelf paper-based circuitry, we fabricated hybrid prototypes of the textbook-embedded clicker and the interactive braille flashcards (Figure 5). During calibration of the sensor, we considered the reproducibility aspects of the sensor, investigated in the previous section, to ensure an appropriate operation of the paper-based clicker. In the case of the textbook clicker, for instance, the student is asked at first-time use of the clicker to locate the answer positions ‘A’, ‘B’ and ‘C’, i.e., ‘$90^{\circ }$’, ‘$45^{\circ }$’ and ‘$0^{\circ }$’, and to browse repeatedly between several combinations of the answer positions, such as (‘A’ → ‘B’ → ‘C’), (‘C’ → ‘B’ → ‘A’), (‘C’ → ‘A’ → ‘B’). The collected sensor data allows for the definition of response margins for each answer position. During operation, the answer location is identified by mapping the sensor data to the pre-defined margins. As shown in Figure 5d, we found that the recorded margins of variation of the sensor responses per angle are unique and non-overlapping ensuring an intact operation of the paper-based clicker, similar to the interactive braille flashcards.

## 3. Materials and Methods

### 3.1. Sensor Fabrication

**Figure 6:**
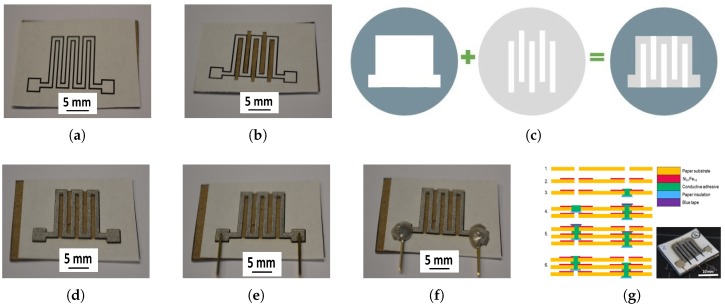
Photographs of (**a**) printed sensor pattern on paper substrates, (**b**) cuts of paper segments corresponding to gaps between inner pattern details of sensor, (**d**) sputter deposition of Ni${}_{81}$Fe${}_{19}$ after alignment of the paper substrate with the shadow mask, (**e**) positioning and fixation of the electrodes, conductive adhesive bonding of electrodes to (**f**) the meander, and (**g**) the sensor stack. The method for patterning a sensor during sputter deposition using the combination of a shadow mask to pattern the outer frame of the meander and micro-machining to cut out inner segments from the paper material, is shown in (**c**).

For the fabrication of paper-based AMR sensors, we used low power magnetron sputter deposition to coat the paper surfaces with a thin film of Ni${}_{81}$Fe${}_{19}$ complying with the hygro-thermal budget of the paper platforms, similarly to what we presented in [15,16]. In particular, the paper substrate has a grammage of 85 $\frac{g}{m^{2}}$, average surface roughness R${}_{a}$ = 2 μm, an average fiber width of 28 μm, and an average segment length (from intersection to intersection) of 100 μm. For its use in clean rooms, the paper fibers are off-the-shelf impregnated with synthetic latex. On the paper substrate we sputter deposited a Ni${}_{81}$Fe${}_{19}$ thin film from a target diameter of 165 mm at 50 Watts, an argon flow of 50 sccm, chamber pressure at sputtering of $10^{-3}$ torr, target-to-substrate distance of 60 mm and magnetron field strength of 200 $\frac{\mathrm{kA}}{m}$. To dry pattern the Ni${}_{81}$Fe${}_{19}$ thin film with highest precision, we used shadow masks magnetically clamped to the paper platform. Additionally, we found that magnetic clamping increases the deposition rate and enhances the magnetic quality of the coating. At last, we pursued the combination of shadow-masking of the coarse outer frame of the pattern and micro-machining of the inner pattern details of the sensor within the paper platform along printed markers (Figure 6a) prior to coating (Figure 6b,c).

After coating, we separated the anisotropic magneto-resistors by paper cutting away from the magnetic thin film. Due to the low thickness of the Ni${}_{81}$Fe${}_{19}$ film, we connected the electrodes to the magneto-resistor by adhesive bonding using an electrically conductive epoxy resin filled with silver flakes. We cured the adhesive pressure-less at room temperature (Figure 6e,f). The roughness of the paper surface, and hence of the paper-based Ni${}_{81}$Fe${}_{19}$ thin film, is advantageous for ensuring a reliable mechanical adhesion between the adhesive bond and the Ni${}_{81}$Fe${}_{19}$ thin film. Lastly, each AMR sensor is composed of a Ni${}_{81}$Fe${}_{19}$ meander with a total of six parallel 10×1 mm${}^{2}$ preferential legs connected at a gap of 1 mm. Excluding the electrodes, the AMR meander spans an area of 11×11 mm${}^{2}$.

Imposed by the paper surface, we found that the fabrication technique and patterning resolution dictate the volume, and therefore the electrical resistance, of the Ni${}_{81}$Fe${}_{19}$ meander. Since miniaturization is not possible, we profited from the thinness of paper, and pursued the fabrication of a sensor stack to achieve larger electrical resistances on the same footprint of a single meander. While ensuring that the meanders are aligned parallel, we stacked several sensors and connected them electrically in series (Figure 6g). As an electrical packaging technique, we used through-paper vias to connect two sensor layers at a time. We manually cut square-shaped vias centered to the probing pads of the single meanders prior to sputter deposition. We used a conductive adhesive resin to fill the through-paper vias. By capping the bottom and top of the via during the filling process, we ensured planarity of the overflow, non-contamination of the neighboring sensor layers and, therefore, electrical insulation. We observed that the adhesive and the cut ends of the paper fibers form a matrix that enhances the reliability of the vias, and that the cured vias eliminate any relative rotation of the sensor layers with respect to each other.

### 3.2. Computational Modeling of Paper-Based Anisotropic-Magneto-Resistance

To interpret the empirically observed differences in the AMR of paper-based Ni${}_{81}$Fe${}_{19}$, we propose a model that captures the macroscopic fibrous nature of the paper surface inherited by the thin Ni${}_{81}$Fe${}_{19}$ coating. In [16], we observed that the paper-based Ni${}_{81}$Fe${}_{19}$ thin film inherits the network topology of (i) fibers of stochastic lengths, widths, thicknesses, and orientations, (ii) crossing each other at so-called lumps and islands, and (iii) surrounded by pores and cavities. Since we are interested in AMR in the film plane, we restrict the model in this work to a perfectly planar paper surface, i.e., the orientation of the interfacial fibers is mapped from a three-dimensional region to a plane. Furthermore, due to the significant difference between the surface roughness of the individual fibers and the surface roughness induced by the fiber network in proportion to the size of the coated paper surface, the surface roughness of the individual fibers is not considered in the model. Last, as long as the coating inherits the fibrous topography, it is assumed that the current can only flow along the fibers since electrical connection to the surrounding is not provided.

**Figure 7:**
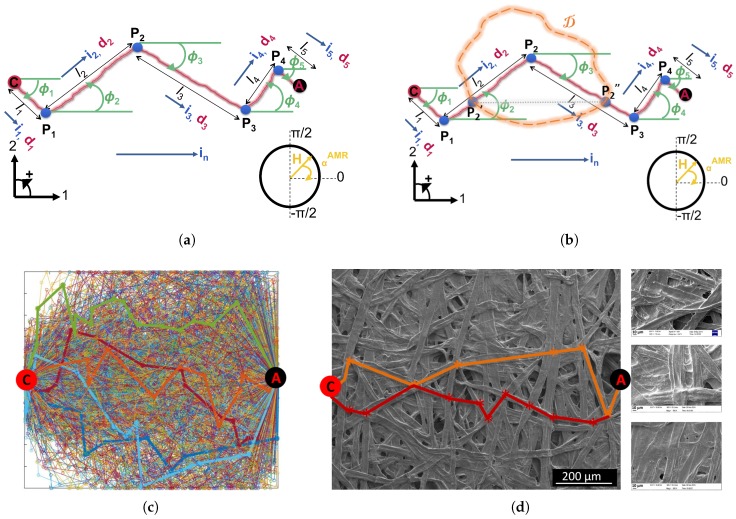
(**a**) Schematic representation of a fiber path (*C*, $P_{i}$, *A*) with nodes $P_{1}$, $P_{2}$, $P_{3}$ and $P_{4}$, segment lengths $\left\{l_{1},l_{2},l_{3},l_{4},l_{5}\right\}$, direction vectors $\{d_{1},d_{2},d_{3},d_{4},$$d_{5}\}$ and angles $\left\{\phi_{1},\phi_{2},\phi_{3},\phi_{4},\phi_{5}\right\}$. $i_{\left\{1,2,3,4,5\right\}}$ is the direction of the electrical current along a segment $i=\left\{1,2,3,4,5\right\}$, and $i_{n}$ is the nominal direction of the electrical current between the cathode and anode. H is the direction of the magnetic field strength, *H*, and $\alpha^{AMR}$ is the anisotropy angle. (**b**) Schematic representation of the trajectory of the electrical current along a fiber path (*C*, $P_{i}$, *A*) crossing a buried region D. ($P_{2}^{{}^{\prime }}$, $P_{2}^{{}^{\prime \prime }}$) represents the shortest direct path along the region D adjoining the fibrous path. (**c**) A computationally obtained statistical sample of 1000 fiber paths between cathode C and anode A with $\phi_{i}$ and $l_{i}$ uniform and Rayleigh distributed, respectively, on a square paper surface. Four fiber paths are highlighted for illustration purposes. (**d**) Left: In comparison to the computational sample, two fiber paths are illustrated on a micrograph of a paper surface. Each fiber path is depicted in a distinct color. Right: Scanning electron microscope graphs of the surface of a clean room paper coated with (from top to bottom): 100 nm, 300 nm and 900 nm of Ni${}_{81}$Fe${}_{19}$.

As for modeling the path of the electrical current across the Ni${}_{81}$Fe${}_{19}$ coating, we propose the geometrical configurations as sketched in Figure 7a,b. Starting at the cathode *C*, the electrical current flows along a possible fiber path (*C*, $P_{i}$, *A*)${}_{j}$, comprised of nodes $P_{i}$ and the center axes of the fiber segments ($P_{i}$, $P_{i+1}$), towards the anode *A*. Each axis of fiber segment is characterized by a length $l_{i}$ and an orientation angle $\phi_{i}$ such that $\phi_{i}=\mathrm{atan}\left(\frac{d_{2}^{i}}{d_{1}^{i}}\right)$ and ${\vec{d}}_{i}=\left(d_{1}^{i},d_{2}^{i}\right)$ is the orientation vector of the fiber segment.

In the presence of a magnetic field, the Ni${}_{81}$Fe${}_{19}$ coating on a fiber segment ($P_{i}$, $P_{i+1}$) is modeled as a single resistor with the corresponding AMR, $R_{i}^{AMR}$. The magneto-resistance of the Ni${}_{81}$Fe${}_{19}$ coating along the fiber path (*C*, $P_{i}$, *A*)${}_{j}$ is the superposition of the AMRs of the proportions of the fiber segments ($P_{i}$, $P_{i+1}$) forming the fiber path, i.e.,

(9) $$R_{j}^{AMR}{|}_{\alpha^{AMR}}=\sum_{i}R_{i}^{AMR}.$$

Given the orientation angle of a fiber segment, $\phi_{i}$, the anisotropy angle for the fiber segment ($P_{i}$, $P_{i+1}$) becomes

(10) $$\alpha_{i}^{AMR}=\alpha^{AMR}-\phi_{i},$$

which is the core of the proposed model, where $\alpha^{AMR}\in \left[0,\pi \right]$ is the nominal anisotropy angle. Hence, the AMR along a fiber segment reads

(11) $$R_{i}^{AMR}{|}_{\alpha^{AMR}}=R_{i}^{⊥}+\left(R_{i}^{∥}-R_{i}^{⊥}\right)\cos^{2}\left(\alpha_{i}^{AMR}\right),$$

and $R_{i}^{⊥}$ and $R_{i}^{∥}$ are the AMRs at the anisotropy angles $\alpha_{i}^{AMR}=0^{\circ }$ and $\alpha_{i}^{AMR}=90^{\circ }$, respectively. Finally, for a planar Ni${}_{81}$Fe${}_{19}$ coating, in which the fibrous topography is inherited, the total magneto-resistance of all parallel fiber segments emanating from the cathode and ending at the anode reads as follows

(12) $$R_{p}^{AMR}{|}_{\alpha^{AMR}}=\frac{1}{\sum_{j}\frac{1}{R_{j}^{AMR}}}.$$

With the increasing Ni${}_{81}$Fe${}_{19}$ thickness, *t*, the electrical current partially passes through direct trails across regions where the fibrous topography becomes buried (Figure 7b,d). Based on parameter β included in the model, the partial elimination of fibrous regions with increasing coating thickness is emulated such that $\beta =\hat{g}\left(t\right)$ is the ratio of areas with fibrous electrical pathways to the total coated paper surface. For any two thicknesses, $t_{1}$ and $t_{2}$,

(13) $$\mathrm{if}\;t_{2}>t_{1},\;\mathrm{then}\;\hat{g}\left(t_{2}\right)\leq \hat{g}\left(t_{1}\right).$$

In dependence of β, the resulting AMR, $R_{t}$, is modeled as a series of the AMRs of the fibrous and non-fibrous regions, D, along the electrical current path, $R_{p}^{AMR}$ and $R_{s}^{AMR}$, respectively, such that

(14) $$R_{t}^{AMR}\left(\alpha^{AMR},\beta \right)=\beta R_{p}^{AMR}{{|}_{\alpha^{AMR}}+\left(1-\beta \right)R_{s}^{AMR}|}_{\alpha^{AMR}}.$$

The AMR of the regions, D, is given by

(15) $$R_{s}^{AMR}\left(\alpha^{AMR}\right)=R_{i}^{s,⊥}+\left(R_{i}^{s,∥}-R_{i}^{s,⊥}\right)\cos^{2}\left(\alpha^{AMR}\right),$$

where $R_{i}^{s,∥}$ and $R_{i}^{s,⊥}$ are the AMRs at the anisotropy angles $\alpha^{AMR}=0^{\circ }$ and $\alpha^{AMR}=90^{\circ }$, respectively.

Without loss of generality, and since we are interested in the qualitative response of paper-based Ni${}_{81}$Fe${}_{19}$ in dependence on the anisotropy angle, the change of AMR of a thin film of Ni${}_{81}$Fe${}_{19}$, i.e., $R_{i}^{s,∥}$, $R_{i}^{∥}$, $R_{i}^{s,⊥}$ and $R_{i}^{⊥}$, and the geometrical dimensions, i.e., $l_{i}$ and the coating surface area, are at first consistently expressed in the model in arbitrary units. For the study of percentage change of AMR, the following empirically obtained relative maximum sensitivities, $\Delta R_{i}^{s}$, of 900 nm of sputtered Ni${}_{81}$Fe${}_{19}$ on a smooth, rigid substrate at room temperature of $\{\frac{7}{12}$, 1, $\frac{7}{12}$ and $-\frac{1}{3}\}\%$ for {−45, 0, 45 and ${90\}}^{\circ }$ are adopted. We first observed the orientation of the interfacial fibers, $\phi_{i}$, through scanning electron micrographs. Taking into consideration that the electrical current flows forward from cathode to anode, we propose that the orientation of the fiber segments along a fiber path (*C*, $P_{i}$, *A*) is to be distributed statistically within $\left[-90^{\circ },90^{\circ }\right]$ (Figure 7). Furthermore, we propose that the length of a fiber segment, $l_{i}$, should statistically vary within [0,1]$l_{i}^{\max}$, where $l_{i}^{\max}$ is the maximum possible length of a fiber segment. To reproduce a fiber path, we assume that both $\phi_{i}$ and $l_{i}$ can take any value at equal likeliness within the definition intervals and, hence, that they are uniformly distributed (*u*). Based on the central limit theorem, $\phi_{i}$ and $l_{i}$ can also be modeled using a normal-like distribution (*n*) that can in turn be represented by an exponential-like distribution (ϵ) or a Rayleigh-like distribution (*r*). In summary, we considered four normalized statistical distributions, $f_{\chi }^{\phi_{i}}$ and $f_{\chi }^{l_{i}}$, for modeling $\phi_{i}$ and $l_{i}$ for χ∈{u,n,ϵ,r}. Based on fiber segment populations of a statistically meaningful size, let’s say 1000, that we generated in a Monte Carlo approach using different combinations of statistical distributions, we concluded that the parameter pair ($\phi_{i}$, $l_{i}$) is best modeled, and hence, the fiber paths are best emulated, using the distribution pair ($f_{u}^{\phi_{i}}$, $f_{r}^{l_{i}}$), which is ultimately adopted in the definition of the electro-magnetic computational model (Figure 7c), and in particular,

(16) $$f_{u}^{\phi_{i}}\left(\phi \right)=\left\{\begin{array}{c} \frac{1}{180}\;,\;\mathrm{if}\;\phi \in \left[-90^{\circ },90^{\circ }\right],\\ 0\;,\;\mathrm{otherwise},\; \end{array}\right.,$$

and,

(17) $$f_{r}^{l_{i}}\left(l\right)=\left\{\begin{array}{c} \frac{1}{\left(1-e^{-\frac{{\left(l_{i}^{\max}\right)}^{2}}{2{\left(\sigma_{l}^{r}\right)}^{2}}}\right)}\frac{l}{{\left(\sigma_{l}^{r}\right)}^{2}}e^{-\frac{l^{2}}{2{\left(\sigma_{l}^{r}\right)}^{2}}}\;,\;\mathrm{if}\;l\in \left[0,l_{i}^{\max}\right],\\ 0\;,\;\mathrm{otherwise}, \end{array}\right.$$

where $\sigma_{l}^{r}=0.43015$, and $l_{i}^{\max}$ is the maximum fiber length as defined above.

Thus, we generated each electrical path one fiber segment at a time. A fiber segment, *i*, is characterized by $\phi_{i}$ and $l_{i}$ and connects at the previous fiber segment i−1. Last, we imposed that the electrical path deterministically starts at the cathode and ends at the anode.

The results obtained with the presented computational model are referred to in Section 2.1.

### 3.3. Sensor Characterization

**Figure 8:**
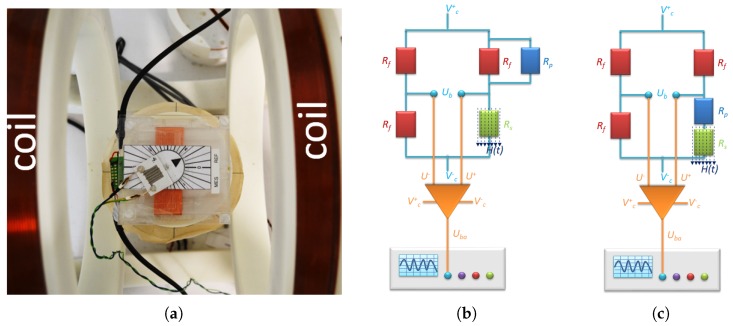
Placement of the sensor fixed on a rotating volvelle within the homogeneity region of the Helmholtz excitation coils (**a**). Schematic of the metrology circuit for determining the resistance of an anisotropic magneto-resistive element $R_{s}$ under the application of an AC magnetic field H(t) for $R_{s}$ with (**b**) large and (**c**) small base resistances.

We used an AC magnetic field produced by Helmholtz coils at a frequency of 50 Hz with a maximum field intensity of 14 $\frac{\mathrm{kA}}{m}$ and a homogeneity region of 40 × 40 mm${}^{2}$. Within the AC magnetic field, we integrated the paper-based sensor with the AMR, $R_{s}$, in a Wheatstone bridge (WB), composed of three resistors with a fixed resistance $R_{f}=$10,000 Ω and an adjustable voltage divider connected in parallel to one of the fixed resistors (Figure 8b). In the case of low-ohmic (<100 Ω) magneto-resistive elements, we used the metrology circuit with a fixed resistance $R_{f}=100$Ω depicted in Figure 8c to ensure the flow of low electrical currents through R${}_{s}$.

We apply a constant voltage, i.e., $V=V_{c}^{+}-V_{c}^{-}=5-\left(-5\right)=10$ Volts at the ends of the WB. In series with the WB circuit, we use an instrumentation amplifier with an adjusted voltage gain γ=10.88 to amplify the WB voltage $U_{b}$ to an oscillograph, Tektronix TDS 2014 such that

(18) $$U_{ba}=\gamma U_{b}=\gamma \left(U^{+}-U^{-}\right),$$

where $U^{+}$ and $U^{-}$ are the input voltages, and $U_{ba}$ is the output voltage of the amplifier. The resistance of the anisotropic magneto-resistive element changes with the strength of the magnetic field and the angular position, $\alpha^{AMR}$, of $R_{s}$ with respect to the magnetic field as follows

(19) $$R_{s}=R_{s}^{c}+R_{s}^{H}\left(H,\alpha^{AMR}\right),$$

where $R_{s}^{c}$ is the resistance at zero magnetic field, *H* is the density of the magnetic field and $R_{s}^{H}$ denotes the varying component of the total resistance. Here, the density of the magnetic field, *H*, is measured through an induced voltage in a measurement coil placed in the proximity of the sensor. While the orientation of the external applied magnetic field is fixed, we rotate the AMR element within the field (Figure 8a). At the balanced state of the WB and in the absence of an external magnetic field, we adjust the resistance of the potentiometer $R_{p}$ such that the circuit exhibits zero voltage ($U_{ba}$ = $U_{b}$ = 0). In the presence of a magnetic field, the resistance of the magneto-resistive element changes ($U_{ba}\neq 0$) and constitutes the unknown resistance in the WB. Obtained from the left and right arms of the WB, the ratio between the input voltage *V* and the input voltages of the amplifier $U^{+}$ and $U^{-}$ are given as

(20) $$U^{+}=\frac{\frac{R_{p}R_{f}}{R_{p}\;+\;R_{f}}}{R_{s}\;+\;\frac{R_{p}R_{f}}{R_{p}\;+\;R_{f}}}V=\frac{R_{p}R_{f}}{R_{s}\left(R_{p}+R_{f}\right)\;+\;R_{p}R_{f}}V,$$

and, $U^{-}=\frac{1}{2}V$, in the case of the first metrology circuit.

For the second metrology circuit, the input voltage $U^{+}$ of the amplifier reads

(21) $$U^{+}=\frac{R_{f}}{R_{s}+R_{p}+R_{f}}.$$

In the case of a balanced WB and in the absence of a magnetic field, i.e., $U_{ba}=0$, we obtain $U^{+}=U^{-}$. Hence, depending on the type of the metrology circuit, the resistance of the potentiometer is given by

(22) $$R_{p}=\frac{R_{s}^{c}R_{f}}{R_{f}-R_{s}^{c}}$$

and

(23) $$R_{p}=R_{f}-R_{s}^{c}$$

for the first and second metrology circuits, respectively. We set $R_{p}$ once per magneto-resistive element to remain valid at all magnetic field intensities and anisotropy angles.

In the case of the presence of a magnetic field, we obtain $U^{+}\neq U^{-}$, and the oscillograph reports a non-zero voltage $U_{ba}$ obtained by DC coupling of the sensor to the oscillograph. In Section 2.1 (Figure 2), we do not consider the steady component of the voltage and investigate the changing component of the sensor signal by AC coupling of the sensor to the oscillograph.

Depending on the type of the metrology circuit, the magneto-resistive element possesses the following resistance:

(24) $$R_{s}=\frac{\left(\gamma V-2U_{ba}\right)R_{p}R_{f}}{\left(\gamma V+2U_{ba}\right)\left(R_{p}+R_{f}\right)}$$

and

(25) $$R_{s}=R_{f}\left(\frac{\gamma V-2U_{ba}}{\gamma V+2U_{ba}}\right)-R_{p}$$

for the first and second metrology circuits, respectively. Then, we calculate the change in resistance due to the presence of a magnetic field as follows:

(26) $$R_{s}^{H}\left(H,\alpha^{AMR}\right)=\frac{\left(\gamma V-2U_{ba}\right)R_{p}R_{f}}{\left(\gamma V+2U_{ba}\right)\left(R_{p}+R_{f}\right)}-R_{s}^{c}$$

and

(27) $$R_{s}^{H}\left(H,\alpha^{AMR}\right)=R_{f}\left(\frac{\gamma V-2U_{ba}}{\gamma V+2U_{ba}}\right)-R_{p}-R_{s}^{c}$$

for the first and second metrology circuits, respectively.

We conducted the characterizations at 21 ${}^{\circ }$C with an ambient relative humidity in the range of 40–70%. We stored the samples between fabrication and characterization in the same measurement conditions.

### 3.4. Application Set-Up

We prototyped both sensor applications using the open-source micro-controller hardware and software platform Arduino UNO. We fed the sensor analog signal to the Arduino platform using the single-supply non-inverting operational amplifier Texas Instruments LM324. We used a plug-in 5V power source external to the Arduino hardware and amplifier circuit, and hence, noise-free. As for other sources of high and low frequency noise, we employed capacitors and cable twisting. For prototyping purposes, we employed the same magnetic field and the Helmholtz coils used in the characterization of the sensor. We note, however, that the source of magnetic field needs to be replaced by a different source of small size and light weight such as a permanent magnet or an inductive charger for a practical applicability of the sensor. Furthermore, we ensured that the magnetic field does not interfere with the functionality of the operational amplifier circuit and the Arduino platform. To identify the angular position of the sensor, we analyzed the sensor digital data using a sketch implemented on the Arduino development environment. We used the Processing environment to handle multimedia tasks needed for the application, for instance the audible locator indications needed for the interactive braille flashcards. We used a single non-bridged sensor, and hence, we solely considered the AC component of the sensor signal by filtering the steady part of the signal by means of a voltage subtraction circuit element designed before amplification. We prompted the user through the serial monitor of the Arduino development environment. For precise handling, we mounted the paper-based sensor on a turnstile that can be rotated using an attached handling knob.

## 4. Summary

In this work, we developed a paper-based AMR sensor that can be used, for instance, in educational applications. We achieved a maximum AMR sensitivity of 0.4% in comparison to an AMR sensitivity of 1% that could be achieved with a Ni${}_{81}$Fe${}_{19}$ thin film prepared under similar conditions on a highly smooth and planar fused silica substrate. Despite the lower sensitivity, we were able to show that the amplified signals of the paper-based sensor in dependence on the anisotropy angles are clearly distinguishable from each other, and hence, usable for the proposed applications.

For a single AMR sensor, we could not make use of the mechanical bendability of the paper substrate as it moves the path of the electrical current away from the magnetization plane, and therefore, deteriorates the AMR sensitivity. For future work, we plan to investigate the foldability of paper for multi-plane sensing. In particular, we will investigate the planar fabrication of AMR sensors on the faces of a polyhedra, at first as a sheet of paper that we will ultimately fold to the desired polyhedral shape. As an application for the paper-based multi-plane sensor, we will consider a paper-based robotic building block.

In this work, we fabricated hybrid prototypes of two educational applications using the paper-based AMR sensor. The lack of off-the shelf paper-based circuitry prevented the natural integration of the paper-based sensor in the application platforms, a book, and a flashcard, and hence, demolished the benefit of the low thickness of the paper-based sensor. As an outlook to the future of all-paper devices, it remains crucial to develop technologies, and make them widely accessible, to power and connect these devices in a way that is as mobile, thin, lightweight, and mechanically bendable as paper itself.

Finally, the paper-based AMR sensor may represent an alternative to potentiometer sensors. For instance, rotary potentiometers rely on the mechanical force between a wiper and a resistive strip to change resistance, which need to be precisely controlled. In comparison, the functional paper-based Ni${}_{81}$Fe${}_{19}$ layer remains untouched during rotation, and the sensing principle is contactless. In terms of size, the surface area of state-of-the-art rotary membrane potentiometers is 10 times larger than the surface area of the Ni${}_{81}$Fe${}_{19}$ thin film used in the paper-based sensor.

## Abbreviations

The following abbreviations are used in this manuscript:

Ni${}_{81}$Fe${}_{19}$	81% of nickel and 19% of iron, permalloy
Ni	Nickel
Fe	Iron
AMR	anisotropic magneto-resistance
AC	alternating current
DC	direct current
WB	wheatstone bridge

## References

[1] Mazzeo A.D., Kalb W.B., Chan L., Killian M.G., Bloch J.F., Mazzeo B.A., Whitesides G.M. (2012). Paper-Based, Capacitive Touch Pads. Adv. Mater..

[2] Xie J., Chen Q., Suresh P., Roy S., White J.F., Mazzeo A.D. (2017). Paper-based plasma sanitizers. Proc. Natl. Acad. Sci. USA.

[3] Kawahara Y., Hodges S., Cook B.S., Zhang C., Abowd G.D. (2013). Instant Inkjet Circuits: Lab-based Inkjet Printing to Support Rapid Prototyping of UbiComp Devices. Proceedings of the 2013 ACM International Joint Conference on Pervasive and Ubiquitous Computing, UbiComp ’13.

[4] Fortunato E., Correia N., Barquinha P., Pereira L., Goncalves G., Martins R. (2008). High-Performance Flexible Hybrid Field-Effect Transistors Based on Cellulose Fiber Paper. IEEE Electron Device Lett..

[5] Stepanov A., Saukkonen E., Piili H. Possibilities of Laser Processing of Paper Materials. Proceedings of the 15th Nordic Laser Materials Processing Conference, Nolamp 15.

[6] Happonen A., Stepanov A., Piili H., Salminen A. Innovation Study for Laser Cutting of Complex Geometries with Paper Materials. Proceedings of the 15th Nordic Laser Materials Processing Conference, Nolamp 15.

[7] Qi J., Buechley L. Electronic Popables: Exploring Paper-Based Computing through an Interactive Pop-Up Book. Proceedings of the Fourth International Conference on Tangile, Embedded and Embodied Interaction.

[8] Siegel A.C., Phillips S.T., Dickey M.D., Lu N., Suo Z., Whitesides G.M. (2010). Foldable Printed Circuit Boards on Paper Substrates. Adv. Funct. Mater..

[9] Martinez R.V., Fish C.R., Chen X., Whitesides G.M. (2012). Elastomeric origami: Programmable paper-elastomer composites as pneumatic actuators. Adv. Funct. Mater..

[10] Kim J., Yun S., Ounaies Z. (2006). Discovery of Cellulose as a Smart Material. Macromolecules.

[11] Chia C., Zakaria S., Ahmad S., Abdullah M., Jani S.M. (2006). Preparation of magnetic paper from kenaf: Lumen loading and in situ sythesis method. Am. J. Appl. Sci..

[12] Ding Z. (2011). Ferrofluid-Impregnated Paper Actuators. J. Microelectromech. Syst..

[13] Li X., Zwanenburg P., Liu X. (2013). Magnetic timing valves for fluid control in paper-based microfluidics. Lab Chip.

[14] Ogata M., Fukumoto M. FluxPaper: Reinventing Paper with Dynamic Actuation Powered by Magnetic Flux. Proceedings of the Computer Human Interaction Conference.

[15] Chen J., Akin M., Yang L., Jiao L., Cheng F., Lu P., Chen L., Liu D., Zhu H. (2016). Transparent Electrode and Magnetic Permalloy Made from Novel Nanopaper. ACS Appl. Mater. Interfaces.

[16] Akin M., Steggeman M., Rissing L. (2016). Paper-based magnetics: Matching paper with permalloy. Cellulose.

[17] Kuijk K., van Gestel W., Gorter F. (1975). The barber pole, a linear magnetoresistive head. IEEE Trans. Magn..

[18] Ger R.R., Xu Y.R., Huang H.T., Wei Z.H. (2012). A permalloy zigzag structure based magnetic bio-sensor. J. Appl. Phys..

[19] Da Camara Santa Clara Gomes T., Medina J.D.L.T., Lemaitre M., Piraux L. (2016). Magnetic and Magnetoresistive Properties of 3D Interconnected NiCo Nanowire Networks. Nanoscale Res. Lett. Nano Express.

[20] Choe G., Steinback M. (1999). Surface roughness effects on magnetoresistive and magnetic properties of NiFe thin films. J. Appl. Phys..

[21] Hood R.Q., Falicov L.M., Penn D.R. (1994). Effects of interfacial roughness on the magnetoresistance of magnetic metallic multilayers. Phys. Rev. B.

[22] Sung G., Shalyguina E.E., Shin K.H. (1999). Theoretical interpretation of positive magnetoresistance in permalloy film. J. Magn..

